# The Representation of Stimulus Features during Stable Fixation and Active Vision

**DOI:** 10.1523/JNEUROSCI.1652-24.2024

**Published:** 2025-01-29

**Authors:** Caoimhe Moran, Philippa A. Johnson, Hinze Hogendoorn, Ayelet N. Landau

**Affiliations:** ^1^ Melbourne School of Psychological Sciences, The University of Melbourne, Parkville, Melbourne, Victoria 3052, Australia; ^2^ Departments of Psychology, The Hebrew University of Jerusalem, Mount Scopus, Jerusalem 9190501, Israel; ^3^ Cognitive and Brain Sciences, The Hebrew University of Jerusalem, Mount Scopus, Jerusalem 9190501, Israel; ^4^ Cognitive Psychology Unit, Institute of Psychology & Leiden Institute for Brain and Cognition, Leiden University, Leiden 2333 AK, Netherlands; ^5^ School of Psychology and Counselling, Queensland University of Technology, St Lucia, Brisbane, Queensland 4072, Australia; ^6^Department of Experimental Psychology, University College London, London WC1H 0AP, United Kingdom

**Keywords:** EEG, feature remapping, feature representation, MVPA, saccades, spatial frequency

## Abstract

Predictive updating of an object's spatial coordinates from presaccade to postsaccade contributes to stable visual perception. Whether object features are predictively remapped remains contested. We set out to characterize the spatiotemporal dynamics of feature processing during stable fixation and active vision. To do so, we applied multivariate decoding methods to EEG data collected while human participants (male and female) viewed brief visual stimuli. Stimuli appeared at different locations across the visual field at either high or low spatial frequency (SF). During fixation, classifiers were trained to decode SF presented at one parafoveal location and cross-tested on SF from either the same, adjacent, or more peripheral locations. When training and testing on the same location, SF was classified shortly after stimulus onset (∼79 ms). Decoding of SF at locations farther from the trained location emerged later (∼144–295 ms), with decoding latency modulated by eccentricity. This analysis provides a detailed time course for the spread of feature information across the visual field. Next, we investigated how active vision impacts the emergence of SF information. In the presence of a saccade, the decoding time of peripheral SF at parafoveal locations was earlier, indicating predictive anticipation of SF due to the saccade. Crucially, however, this predictive effect was not limited to the specific remapped location. Rather, peripheral SF was correctly classified, at an accelerated time course, at all parafoveal positions. This indicates spatially coarse, predictive anticipation of stimulus features during active vision, likely enabling a smooth transition on saccade landing.

## Significance Statement

Maintaining a continuous representation of object features across saccades is vital for stable vision. In order to characterize the spatiotemporal dynamics of stimulus feature representation in the brain, we presented stimuli at a high and low spatial frequency (SF) at multiple locations across the visual field. Applying EEG-decoding methods we tracked the neural representation of SF during both stable fixation and active vision. Using this approach, we provide a detailed time course for the spread of feature information across the visual field during fixation. In addition, when a saccade is imminent, we show that peripheral SF is predictively represented in anticipation of the postsaccadic input.

## Introduction

The early visual system encodes the world retinotopically: objects are projected to early visual areas with spatial coordinates determined by their position on the retina. This complicates accurate localization, given that eye movements continuously shift the retinal position of objects. Despite this, visual perception remains largely unperturbed. There is a general consensus that predictive remapping of a stimulus’ spatial coordinates from their retinotopic presaccadic to postsaccadic position contributes to visual stability (Mays and Sparks, 1980; Bruce and Goldberg, 1985; Duhamel et al., 1992; Walker et al., 1995; Thompson et al., 1996; Umeno and Goldberg, 1997; Gottlieb et al., 1998; Nakamura and Colby, 2002; Sommer and Wurtz, 2002; Merriam et al., 2007; Parks and Corballis, 2008; Knapen et al., 2016; Golomb, 2019; Moran et al., 2024). We recently investigated the temporal unfolding of spatial remapping in humans (Moran et al., 2024). Using EEG in combination with multivariate pattern analysis (MVPA) to provide high temporal precision, we found evidence for the stimulus at the remapped location when the stimulus was presented in a short time window before saccade onset. In order to track and identify changes in visual input across saccades, anticipation of postsaccadic stimulus features may also be important, with some suggesting that stimulus identity is relayed from presaccadic to postsaccadic neurons (Gordon et al., 2008; Hollingworth et al., 2008; Melcher and Colby, 2008). Whether predictive remapping is a feature selection process is a highly contentious issue (Cavanagh et al., 2010; Harrison et al., 2013; He et al., 2018).

The majority of studies investigating the transfer of featural information across saccades in humans are behavioral. For example, studies have demonstrated feature remapping using crowding (Harrison et al., 2013) and the tilt aftereffect (TAE; Gibson, 1937; Gibson and Radner, 1937) demonstrating a transfer of feature information to the postsaccadic retinotopic location when a saccade is imminent (Melcher, 2005, 2009; Zimmermann et al., 2017). A major issue with these studies however is the limited sampling of spatial locations, making it difficult to determine if TAE and crowding effects are unique to the remapped location or more widespread. Studies investigating illusory aftereffects during stable fixation have found that an adaptor presented at one location induced an aftereffect evenly across an array of locations tested, perhaps capturing feature-based attention (FBA) which acts across the entire visual field (Liu and Hou, 2011; Liu and Mance, 2011). In addition, the stimulus to be remapped is often the saccade target, meaning the remapped location overlaps with the fovea (Herwig and Schneider, 2014; Herwig, 2015; Paeye et al., 2017; Fabius et al., 2019). This makes it difficult to rule out fovea-specific processes as an explanation for effects at the remapped position. There is plenty of evidence from psychophysics (Fan et al., 2016; Yu and Shim, 2016; Weldon et al., 2020), neuroimaging (Williams et al., 2008; Fan et al., 2016), and brain stimulation (Chambers et al., 2013) to suggest that the fovea contributes to the processing of peripheral stimuli during stable fixation. In addition, recent behavioral research reported foveal feedback under saccade conditions (Kroell and Rolfs, 2022).

Invasive neurophysiological recordings in animals and neuroimaging methods in humans have demonstrated that neurons in the LIP, SC, FEF, and extrastriate cortex exhibit remapping characteristics (Duhamel et al., 1992; Walker et al., 1995; Umeno and Goldberg, 1997; Nakamura and Colby, 2002; Merriam et al., 2003, 2007; Mirpour and Bisley, 2012, 2016; Zirnsak and Moore, 2014; Wang et al., 2023). Despite this, there is almost no direct neural evidence that the responses recorded at remapped locations contain information about stimulus features. There are two exceptions that offer conflicting results. One animal study demonstrated shape selectivity in the future field of neurons in LIP (Subramanian and Colby, 2014) while another study, recording from area MT in monkeys, found no selectivity for motion direction in the remapped response (Yao et al., 2016). There is even less neurophysiological evidence in humans. That said, transcranial magnetic stimulation (TMS) to the FEF has been shown to interfere with the trans-saccadic memory of stimulus features, perhaps by interrupting the remapping process (Prime et al., 2010).

Given previous evidence that peripheral stimulus information can influence perception and the neural response at unstimulated positions, even in the absence of a saccade, it is important to understand the time course of this process. This can be done by taking a temporally resolved approach to the spread of stimulus information across the visual field. This allows a dissociation between information at the remapped location due to feedback processes that exist even in the absence of a saccade and information transferred to the remapped location as a result of saccadic remapping. In addition, to circumvent confounding fovea-specific effects with effects unique to remapping, the stimulus to be remapped needs to be separated from the saccade target, inducing peripheral-to-peripheral remapping rather than peripheral-to-foveal remapping. Finally, to ensure bottom-up visual input does not interfere with the unfolding dynamics of the remapped representation, a brief stimulus must be used so that no visual input enters the eye at the postsaccadic retinotopic position.

In this study, we conducted a decoding analysis of human EEG data sampling multiple stimulus locations. Participants completed fixation trials and saccade trials during which a grating stimulus, at a high or low spatial frequency (SF), was briefly presented at various locations on the screen. Firstly, to characterize the extent to which SF information spreads across the visual field during stable fixation, we ran a cross-location decoding analysis, training a classifier on a single location and testing it on other locations. To foreshadow the results, we found that SF information spreads from the actual stimulus position across the cortex as a function of eccentricity, ultimately providing a position-invariant representation of SF. Secondly, to determine whether SF is predictively remapped to the postsaccadic retinotopic position, we performed another cross-location decoding analysis, training on fixation trials, and testing on saccade trials. We found that the SF of peripheral stimuli in saccade trials could be decoded earlier at the remapped location than stimuli at the same eccentricity during stable fixation. Importantly, however, by testing incongruent locations we found that this early decoding is not unique to the remapped location but rather signifies a spatially coarse predictive representation of SF.

## Materials and Methods

Participants, experimental design, and data preprocessing were adapted from Moran et al. (2024), and existing data from this paper were reanalyzed here.

### Participants

Participants were required to complete six testing sessions across different days, including one screening session. Thirteen observers took part in the initial screening session, of whom two did not want to continue with the remaining sessions and one was excluded due to noisy data (the result of an incorrect cap size). After exclusion, 10 observers (three male; mean age, 25.7 years; SD, 2.0 years) with normal or corrected-to-normal vision remained. The experimental protocol was approved by the human research ethics committee of The University of Melbourne, Australia (Ethics ID: 2021-12985-16726-4) and conducted in accordance with the Declaration of Helsinki. All observers provided informed consent before beginning the experiment and were reimbursed AU$15 per hour for their time, plus an additional AU$20 after completing all sessions.

### Stimuli and procedure

Stimuli were programmed in MATLAB Version R2020a, using the Psychophysics Toolbox extension (Brainard, 1997; Pelli, 1997; Kleiner et al., 2007). They were presented on an ASUS ROG PG258 monitor (ASUS) with a resolution of 1,920 × 1,080 running at a refresh rate of 120 Hz. Participants were seated in a quiet, dark room with their heads supported by a chin rest, positioned 80 cm from the screen.

Stimuli consisted of sinusoidal gratings presented within a circular Gaussian window [i.e., Gabors; outer diameter, 8° of visual angle (dva); 100% contrast] presented on a gray background for 100 ms. The stimulus could appear at an SF of 0.33 or 1 c/dva while the orientation of the grating was fixed at 0° (except on catch trials). For catch trials, participants were instructed to respond to an oddball grating of a different orientation (90°) which could appear every 11–20 trials. These trials were included in order to ensure attentive processing of the grating stimuli and were excluded from the main analysis.

Two fixation points (0.41 dva), one black and one white, were horizontally aligned and subtended 10.04 dva to the left and right of the screen center (20.08 dva apart). They appeared at the beginning of the experiment and remained visible throughout, excluding breaks. Participants were instructed to fixate on the black fixation point at all times. Periodically during the experiment, a saccade cue appeared: the color of the fixation points gradually changed, such that over a period of 1.2 s the black fixation point became white and vice versa. Participants were instructed to monitor the color of the fixated fixation point and plan and execute a saccade from one fixation point to the other as soon as they detected the color change, such that they were always fixating the black fixation point. A gradual color change was chosen as the saccade cue to minimize cue-induced effects. Abrupt cue onsets can elicit visual evoked potentials (VEPs; Scheel et al., 2015) which may obscure the neural dynamics associated with predictive remapping.

**Figure 1.**
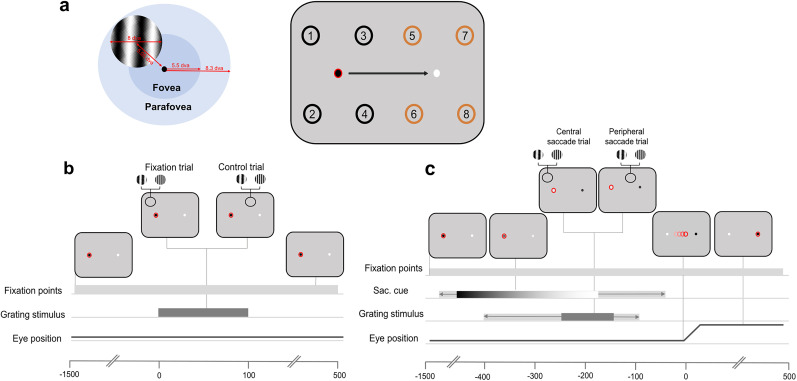
Adapted from Moran et al. (2024). ***a***, Stimulus dimensions and configuration. Left, Stimuli were sinusoidal gratings (diameter, 8 dva) presented at 5.05 dva from a fixation point. Stimuli extended across the foveal and parafoveal regions. Right, Participants always fixated on the black fixation point and made a saccade when cued by a gradual color change to white. Stimuli were presented around fixation on fixation trials (Locations 1–4; black) or around the white fixation point/saccade target on control trials and saccade trials, respectively (Locations 5–8; orange). The red circle around the black fixation point indicates eye position. The numbers on the screen indicate all possible stimulus presentation locations. ***b***, In fixation trials, participants maintained fixation on the black fixation target (shown here with a red ring to indicate the position of the eyes) throughout the trial, never fixating on the white. Approximately 400–800 ms after trial onset, the grating stimulus was presented at one of four locations around fixation at either a low or high SF. The stimulus was displayed for 100 ms. In control trials, participants fixated on the black fixation point. A grating stimulus was presented at one of four locations around the alternate fixation point (white circle). ***c***, In saccade trials, participants fixated on the black fixation point. After 200–600 ms, the black fixation point changed to white and the white fixation point changed to black, which served as the saccade cue. The colors gradually changed over a period of 1.2 s, indicated here by the color gradient. The light gray bar behind the gradient refers to the possible start/end times of the color change. In a period ranging from 400 to 100 ms before saccade onset, a grating stimulus was presented at one of four locations around the current fixation (“central saccade trials”—not analyzed) or the saccade target (“peripheral saccade trials”). The stimulus was presented for 100 ms. The light gray bar behind the dark gray bar refers to the possible start/end times of the stimulus.

Different conditions were determined by the conjunction of fixation position and the location of the grating stimulus. In fixation trials, the fixation was stable (no color change occurs), and stimuli were briefly presented (100 ms) in one of four positions around the current black fixation point (Locations 1–4; Fig. 1*a*, right). In control trials, the fixation was stable, and stimuli were briefly presented (100 ms) on the opposite side of the screen, in one of four locations around the white fixation point (Locations 5–8; Fig. 1*a*, right). In saccade trials, there was a color change, and stimuli appeared at a variable delay after the saccade cue. This delay was adjusted for each participant in order to sample trials with approximately 200 ms between stimulus onset and saccade onset. To do so, the latency between grating onset and saccade onset was recorded on every trial and averaged over the previous 100 trials. In saccade trials, stimuli could appear around current fixation (Fig. 1*c*, central saccade trial) or saccade target (Fig. 1*c*, peripheral saccade trial). Only peripheral saccade trials were analyzed in the current manuscript and will be referred to as “saccade trials” henceforth (see Fig. 1*b*,*c* for task design).

Stimulus locations were separated by 5.05 dva and were also 5.05 dva away from the nearest fixation point. The fovea covers the central 5.5 dva while the parafovea covers 8.3 dva around fixation (Hendrickson, 2005). Our grating stimuli covered 8 dva and therefore, on fixation trials, extended across foveal and parafoveal regions (Fig. 1*a*, left). For simplicity, we refer to these stimuli as parafoveal stimuli. We discuss the implications of this stimulus placement in the discussion section.

Each experimental session contained a total of 2,400 trials: 1,120 fixation trials, 1,120 saccade trials (split evenly between central and peripheral saccade trials), and 160 control trials, randomly interleaved across five blocks. There was a total of 480 trials per block, with a minibreak every 100 trials. Trials were split evenly between fixation and saccade trials (224 trials each) with the remaining allocated to control trials (32 trials).

### EEG and eye-tracking preprocessing

EEG and EOG data were recorded at 2,048 Hz using a BioSemi system, with 64 active electrodes and 6 ocular electrodes. Electrodes were placed using the 10–20 system. The continuous EEG and eye-tracking data were preprocessed offline using MATLAB Version R2020a and EEGLAB toolbox (v2021.0; Delorme and Makeig, 2004). The data were first downsampled to 256 Hz and then rereferenced to the mastoids. Eye-tracking data were recorded using an EyeLink 1000 eye tracker (SR Research) at 1,000 Hz. The eye tracker was calibrated at the start of the experiment and at the beginning of every block. In addition, drift correction was applied at each minibreak within a block. The eye-tracking data were synchronized with the EEG using the EYE-EEG toolbox version 0.81 (Dimigen et al., 2011). Saccades were detected using a velocity-based algorithm (Engbert and Kliegl, 2003) within the EYE-EEG toolbox. Successive eye positions were considered saccades if the velocity of the left eye exceeded a threshold of five standard deviations (median-based) of all recorded eye velocities (excluding blink intervals) for at least 15 ms. A low threshold was used to ensure the detection of microsaccades which are important for the removal of eye movement–related artifacts in later steps (Dimigen et al., 2009). If the time between two saccadic events was <50 ms, only the first saccade was kept, in order to avoid including postsaccadic oscillations as separate saccades (Dimigen, 2020). Following this, saccades were considered valid if they exceeded an amplitude of 15 dva.

The EEG data were then notch filtered at ∼50 Hz to remove electrical artifacts and bandpass filtered between 0.1 and 80 Hz. Automatic data rejection was employed to remove any major artifacts using the artifact subspace reconstruction (ASR) method in EEGLAB. The ASR rejection threshold parameter *k* was set to 15. Bad channels, noted during data collection and confirmed later offline, were spherically interpolated.

To correct for eye movement artifacts in the EEG, we applied independent component analysis (ICA; Makeig et al., 1996). To identify eye movement–related components, the variance ratio of the component activation during periods of eye movements (blinks and saccades) was compared with that during fixation periods (Plöchl et al., 2012). ICA was performed in a separate preprocessing pipeline containing an additional high-pass filter (Hamming windowed sinc FIR; edge of the passband, 2 Hz). It was run on clean continuous data with major movement artifacts removed. The ICA weights were then appended to the corresponding datasets in the original preprocessing timeline, and IC activations were recomputed. Components were rejected if the mean variance of activity selected around a saccade (−0.02 to 0.01 ms) was 10% greater than the mean variance during fixation periods (Plöchl et al., 2012; Dimigen, 2020).

For all trial types, if gaze deviated >2.5 dva from the current fixation point while the stimulus was on the screen, the trial was removed. Similarly, if the saccade landing was >2.5 dva away from the saccade target, the trial was discarded. Applying these criteria, we discarded 8.1% of fixation trials, 9.5% of saccade trials, and 7.5% of control trials. In addition, only trials in which the physical stimulus was removed from the screen by the time the eyes arrived at the saccade target were included. Previous studies of trans-saccadic fusion (Wolf et al., 1980; Jonides et al., 1982) have been challenged because the results could be explained by lingering visual monitor persistence (Irwin et al., 1983, 1990). This is not the case in the present study as the gray-to-gray time of the monitor used was 1 ms. Additionally, it has been reported that LCD monitors are optimal when visual persistence is a concern given the short rise times (1–6 ms; Lagroix et al., 2012).

EEG data were epoched for fixation, control, and saccade trials separately. Across all trial types, epochs were time-locked to the presentation of the grating. Epochs were extracted from 200 ms before stimulus onset to 500 ms after and were baseline corrected to the mean of the 100 ms period before stimulus onset. Fixation trials were subsequently included in the training set for the main analysis.

### Multivariate pattern analysis

All MVPAs were performed in Python (v3.11.3). Each classification analysis was performed at the level of single subjects using the amplitude from the 64 electrode channels as input. For all classification analyses, fixation trial data from each location were used to train time-resolved pairwise linear discriminant analysis (LDA) classifiers (Grootswagers et al., 2017) to dissociate the neural activation patterns associated with the presentation of the stimulus at a high and low SF at a single location. There were on average 1,345 trials in each location-specific training set, split equally between high SF and low SF trials. This means there were approximately 5,380 training trials per participant.

#### Decoding SF during stable fixation

**Figure 2.**
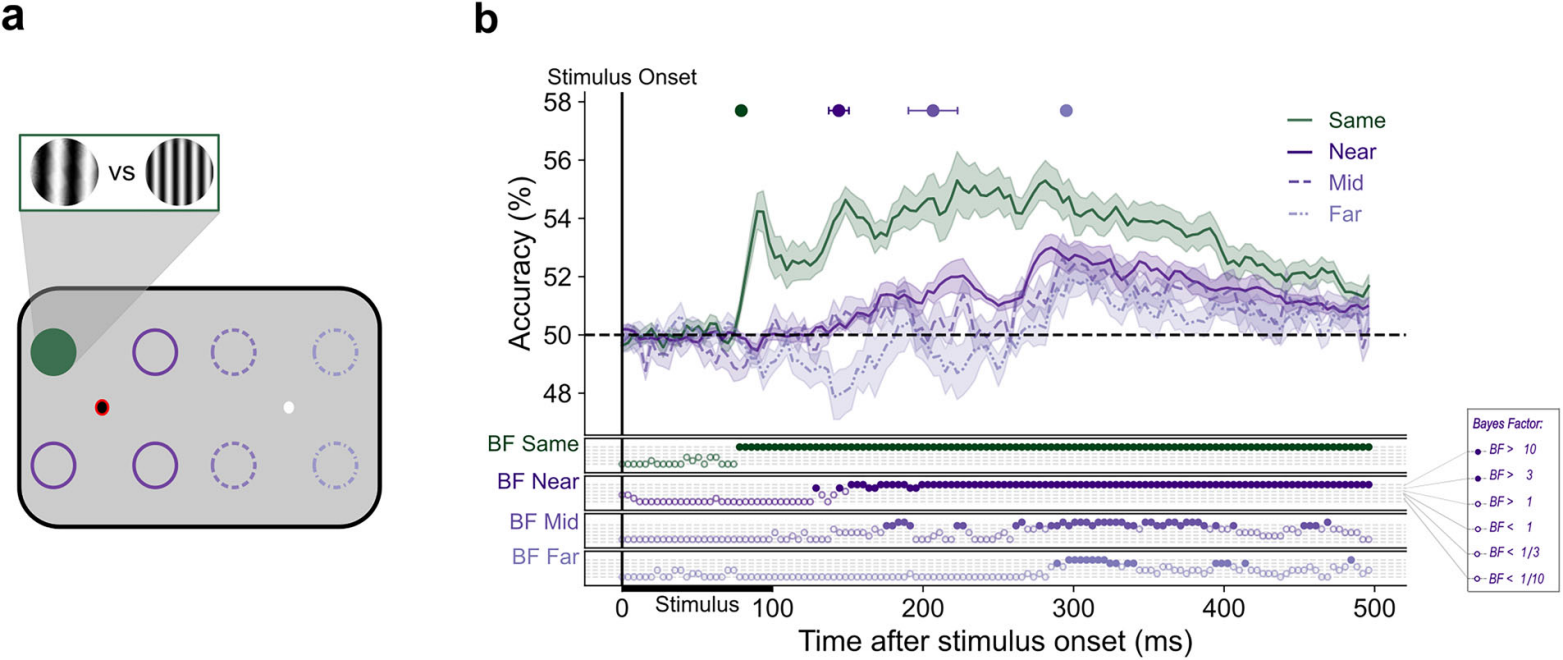
A spatially invariant representation of spatial frequency. Classifiers were trained and tested to distinguish patterns of neural activity evoked by the presentation of a stimulus at different locations on the screen. In panel ***a***, the circles represent possible stimulus presentation locations and one example configuration of train and test locations for classification. The dark green circle represents the location on which the classifier was trained, the dark purple circles indicate the near test locations, the medium purple (dashed) indicates the mid-test locations, and the light purple (dot dashed) indicates the far test locations. The red ring signifies current fixation. The gratings illustrate stimuli at low and high SF. ***b***, The mean classification over time for fixation trials trained and tested at the same location (green line) and trained on one location and tested on near locations (dark purple line), mid locations (medium purple, dashed), and far locations (light purple, dot dashed). The plotted results reflect the average performance at corresponding training and testing timepoints. All decoding results are averaged over locations at the same eccentricity, across left and right fixation, and subsequently, across subjects. Shaded areas depict the standard error of the mean across subjects. Above-chance decoding onsets are plotted with 95% confidence intervals. The Bayes factors (BF) below the plots indicate the timepoints at which there was substantial evidence in favor of the alternative hypothesis, i.e., decoding above-chance (filled circles). Timepoints at which there was not enough evidence or substantial evidence for the null hypothesis, i.e., chance level, are indicated by open circles.

First, to examine the representation of SF during stable fixation, we tested each location-specific LDA classifier on independent fixation trials from the same location using a fivefold cross-validation method. This was done separately for each of the four parafoveal locations (same condition). Fixation could be on the left or the right of the screen meaning a total of eight classifiers were trained and tested. Classification results were then averaged across all locations. Subsequently, we tested whether classifiers trained to dissociate SF at one location could perform above-chance when tested on stimuli at varying eccentricities from the current fixation. To do this, we ran a cross-location decoding analysis. In this analysis, data from one of the four parafoveal locations were used to train a classifier that was subsequently tested on data from each of the remaining seven locations (i.e., fixation and control trials; Fig. 2*a*). Classification results were grouped based on stimulus eccentricity from current fixation and averaged within each eccentricity group: near (∼5 dva), mid (∼10 dva), and far (∼20 dva). This allows us to see the degree to which the representation of SF spreads across the visual field. In other words, how well does the representation of SF at one retinotopic position generalize to another?

#### Decoding SF during active vision

To examine the time course of SF information availability on saccade trials, we ran additional cross-location decoding analyses, training on fixation trial data, and testing on saccade trials. These were carried out time-locked to stimulus onset (0–500 ms after stimulus onset). Only trials in which the saccade occurred in a range between 100 and 400 ms after stimulus onset were included in the analysis. To understand if features are precisely remapped to the postsaccadic retinotopic position, we split saccade trials into “congruent” or “incongruent” depending on the training set location. Congruent trials were those saccade trials in which a stimulus was presented at the same retinotopic position as the position used in the training set, relative to the other fixation point (Fig. 1*a*, right, Locations 1–5, 2–6, 3–7, 4–8). This is because the corresponding remapped location overlaps with the trained location. Incongruent trials included the nonremapped location at the same eccentricity. For example, if the classifier was trained on Location 1, congruent saccade trials would include Location 5 stimuli, and incongruent trials would include Location 6 stimuli (Fig. 1*a*, right). Classifiers were tested on the corresponding timepoints used for training, i.e., the time diagonal.

To examine whether peripheral SF is predictively represented in the presence of a saccade, classifiers were trained on fixation trial data from parafoveal positions. Each location-specific classifier was tested only on trials from the congruent peripheral location using either saccade or control data as the test set. To determine whether SF information is transferred uniquely to the remapped location in the presence of a saccade, we used the same trained fixation classifiers to test peripheral stimuli from congruent versus incongruent locations. Finally, to characterize the representation of SF during a saccade more generally, we cross-tested the location-specific fixation classifiers on saccade trials from near, mid, and far eccentricities. Classification scores were averaged within each eccentricity group. This gives us an understanding of when SF at different eccentricities becomes available at positions around fixation during the perisaccadic period.

### Statistical inference

We used Bayes factors (BFs) to determine above-chance decoding and at-chance decoding (i.e., null hypothesis) at every timepoint within each of the 10 participants using the BayesFactor R package (Morey and Rouder, 2018) implemented in Python (Teichmann et al., 2022). We set the prior for the null hypothesis at 0.5 (chance decoding) for assessing the decoding results and at 0 for assessing differences across decoding results. A half-Cauchy prior was used for the alternative hypothesis with a medium width of *r* = 0.707. Based on Teichmann et al. (2022), we set the standardized effect sizes expected to occur under the alternative hypothesis in a range between −∞ and ∞ to capture above and below-chance decoding with a medium effect size (Morey and Rouder, 2018).

BFs >1 indicate that there is more evidence for the alternative hypothesis than the null hypothesis (Dienes, 2011) with a BF >3 considered as “substantial” evidence for the alternative hypothesis, i.e., decoding above-chance level. A BF <1/3 is considered substantial evidence for the null hypothesis, i.e., chance-level decoding (Jeffreys 1939, 1961; Wetzels et al., 2011).

To determine the onset of above-chance decoding, we found the first three consecutive timepoints where the Bayes factors were >3 (Wetzels et al., 2011). To compare onset times across different conditions, we calculated a 95% confidence interval using a jackknife procedure. This involved iteratively running the Bayes analysis on the decoding time series results, leaving two participants out each time (*n* = 45 permutations for 10 participants). We then took the 95th percentile of the resulting distributions (Grootswagers et al., 2024).

## Results

### Decoding SF across the visual field during stable fixation

#### Same location decoding

Using a fivefold cross-validation procedure, we found that classifiers effectively labeled the test set trials as their correct SF across all locations (Fig. 2*b*, “same”). Above-chance decoding of SF emerged by 79 (79–80) ms after stimulus onset and was sustained throughout the entire trial period. This demonstrates that when training and testing at the same parafoveal location, our classifiers could robustly extract SF information from the EEG signal.

#### Cross-decoding from near eccentric positions

There was substantial evidence for above-chance cross-location decoding of SF at near locations (Fig. 2*a*, dark purple). Above-chance decoding emerged by 144 (137–151) ms, later than when training and testing on the same location (Fig. 2*b*, “near”). This demonstrates that ∼65 ms after SF information is available at the stimulus presentation location, it spreads to adjacent locations at the same eccentricity from fixation. These results indicate that the representation of SF generalizes to nearby parafoveal locations after ∼140 ms.

#### Cross-decoding from mid-eccentric positions

We next wanted to investigate whether SF generalizes to the rest of the visual field or whether it is constrained to locations near the trained location. The same four parafoveal fixation classifiers were tested on trials in which the stimulus was presented 10 dva away from fixation, in the periphery (Fig. 2*a*, medium purple, dashed). Dissociation between high and low SF first rose above chance at 207 (190–223) ms poststimulus onset (Fig. 2*b*, “mid”), indicating an emerging generalization of the SF representation.

#### Cross-decoding from far eccentric positions

Above-chance cross-decoding of SF at far peripheral locations (20 dva; Fig. 2*a*, light purple, dot dashed) emerged later at 295 (293–297) ms poststimulus onset (Fig. 2*b*, “far”). These results indicate an emerging spatially invariant representation of SF that spreads over time from the presented stimulus position across the entire visual field.

### Decoding SF at the remapped location with and without a saccade

As shown in the previous analyses, under stable fixation, SF decoding generalizes across the entire visual field. We therefore expect to find SF information at the “remapped” position even in the absence of a saccade. Notably, however, the temporal availability of SF information at the remapped location should gain a predictive nature under active vision. In other words, the latency of above-chance decoding of SF at the remapped location should be earlier for saccade trials compared with control trials in which no saccade is performed (but stimulus presentation is otherwise equivalent).

**Figure 3.**
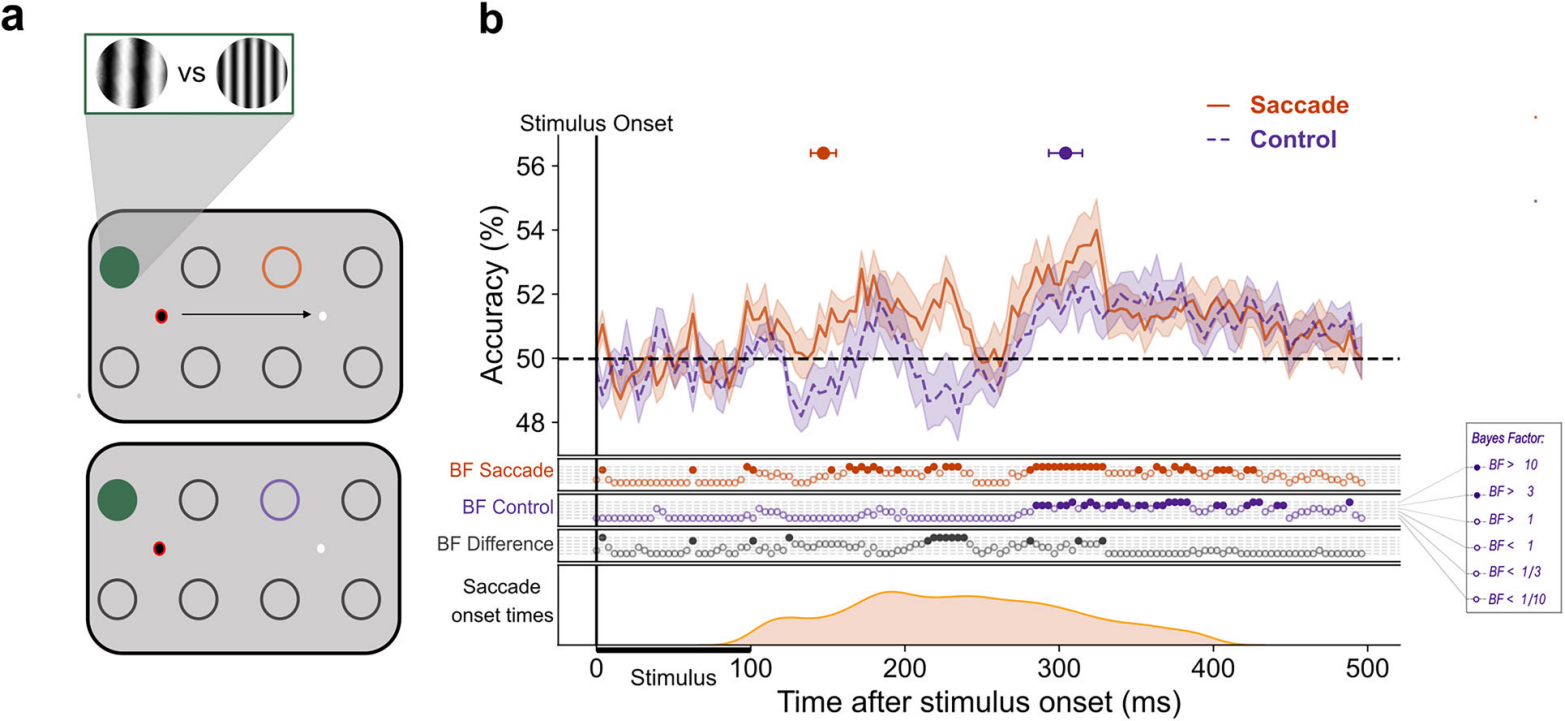
Earlier decoding of SF at the remapped location with a saccade. This figure illustrates the difference between decoding at the remapped location with and without a saccade. In panel ***a***, the circles represent possible stimulus presentation locations and an example configuration of train and test locations for the classification of saccade trials (top panel) and control trials (bottom panel). The green-filled circle represents the location on which the classifier was trained, and the orange and purple circles signify the test locations for saccade and control trials, respectively. The red ring signifies current fixation. The gratings illustrate stimuli at low and high SF. ***b***, The mean classification over time for classifiers trained on fixation trials and tested on either saccade trials (orange) or control (purple) trials. The plotted results reflect the average performance at corresponding training and testing timepoints. All decoding results are averaged over classifiers, across left and right fixation, and subsequently, across subjects. The shaded areas depict the standard error of the mean across subjects. Above-chance decoding onsets are plotted with 95% confidence intervals. The Bayes factors (BF) below the plots indicate the timepoints at which there was substantial evidence in favor of the alternative hypothesis, i.e., decoding above-chance (filled circles). Timepoints at which there was not enough evidence or substantial evidence for the null hypothesis, i.e., chance level, are indicated by open circles. The distribution of saccade onset times is plotted below.

From 147 (139–155) ms poststimulus onset in saccade trials, the classifier was able to predict above-chance which SF was presented in the periphery. This is in contrast to decoding of peripheral trials in the absence of a saccade (i.e., control) where peripheral SF could be reliably decoded only ∼150 ms later, at 304 (293–315) ms poststimulus onset (Fig. 3*b*). Substantial differences in the decoding time courses between conditions emerged at 185 (172–197) ms poststimulus onset and was maintained until ∼238 ms. These results indicate that in the presence of a saccade, information about SF is available at the remapped location earlier than during stable fixation. This finding is consistent with the predictive remapping of SF as a result of the saccade.

### Predictive representation of SF is spatially coarse

The previous analysis showed initial evidence of predictive remapping of peripheral stimulus SF to the remapped location. Next, we wanted to determine the extent to which this predictive representation is spatially precise.

**Figure 4.**
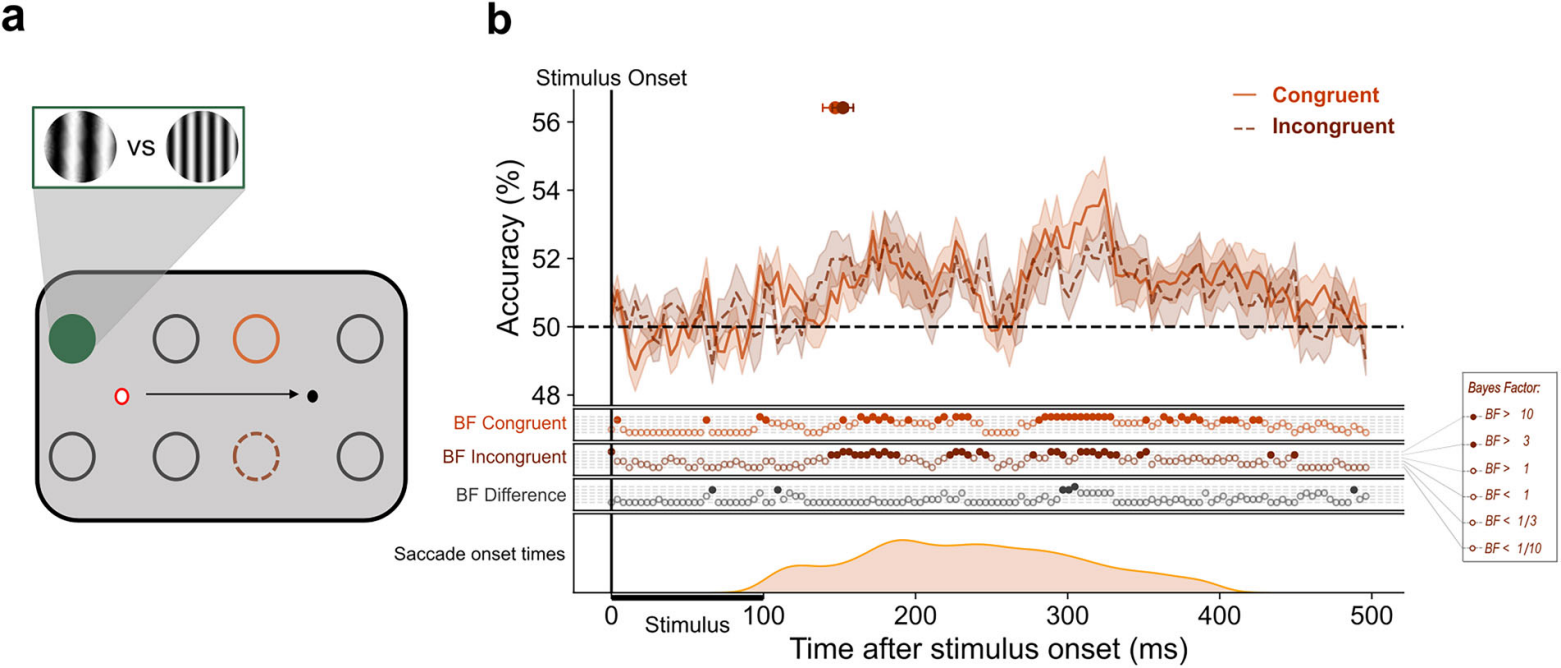
No spatially precise remapping of SF. ***a***, Classifiers were trained on high- versus low-frequency stimuli presented at each of the four locations around current fixation (red ring) producing four training sets. Illustrated is an example configuration of train and test locations for classification. The green-filled circle represents the location on which the classifier was trained. Each training set was paired with two test sets. One test set in which stimuli were presented at a congruent location (light orange, solid line), and another in which stimuli were presented at an incongruent location (dark orange, dashed line) in the periphery. The black arrow represents the saccade. The gratings illustrate stimuli at low and high SF. ***b***, The mean classification over time for congruent trials (light orange, solid line) and incongruent trials (dark orange, dashed line) in saccade trials. The plotted results reflect the average performance at corresponding training and testing timepoints. The shaded areas depict the standard error of the mean across subjects. All decoding results are averaged across left and right fixation, and subsequently, across subjects. Above-chance decoding onsets are plotted with 95% confidence intervals. The Bayes factors (BF) below the plots indicate the timepoints at which there was substantial evidence in favor of the alternative hypothesis, i.e., decoding above-chance (filled circles). Timepoints at which there was not enough evidence or substantial evidence for the null hypothesis, i.e., chance level, are indicated by open circles. The distribution of saccade onset times is plotted below.

We found that SF could be decoded from both the congruent and incongruent locations at 147 (139–155) ms and 152 (145–159) ms, respectively, with no reliable evidence for any difference between decoding at these locations (Fig. 4*b*). This suggests that the ability to decode SF from peripheral positions cannot be explained by a precise transfer of stimulus features to the postsaccadic retinotopic position. Rather, decoding is more spatially coarse such that each parafoveal classifier (Fig. 4*a*, green-filled circle) can decode SF from all peripheral positions. Thus, we can conclude from this analysis that while the saccade allows predictive anticipation of peripheral stimulus features, this response does not contain information about the postsaccadic retinotopic position of the stimulus.

### Representation of peripheral spatial frequency at parafoveal positions

**Figure 5.**
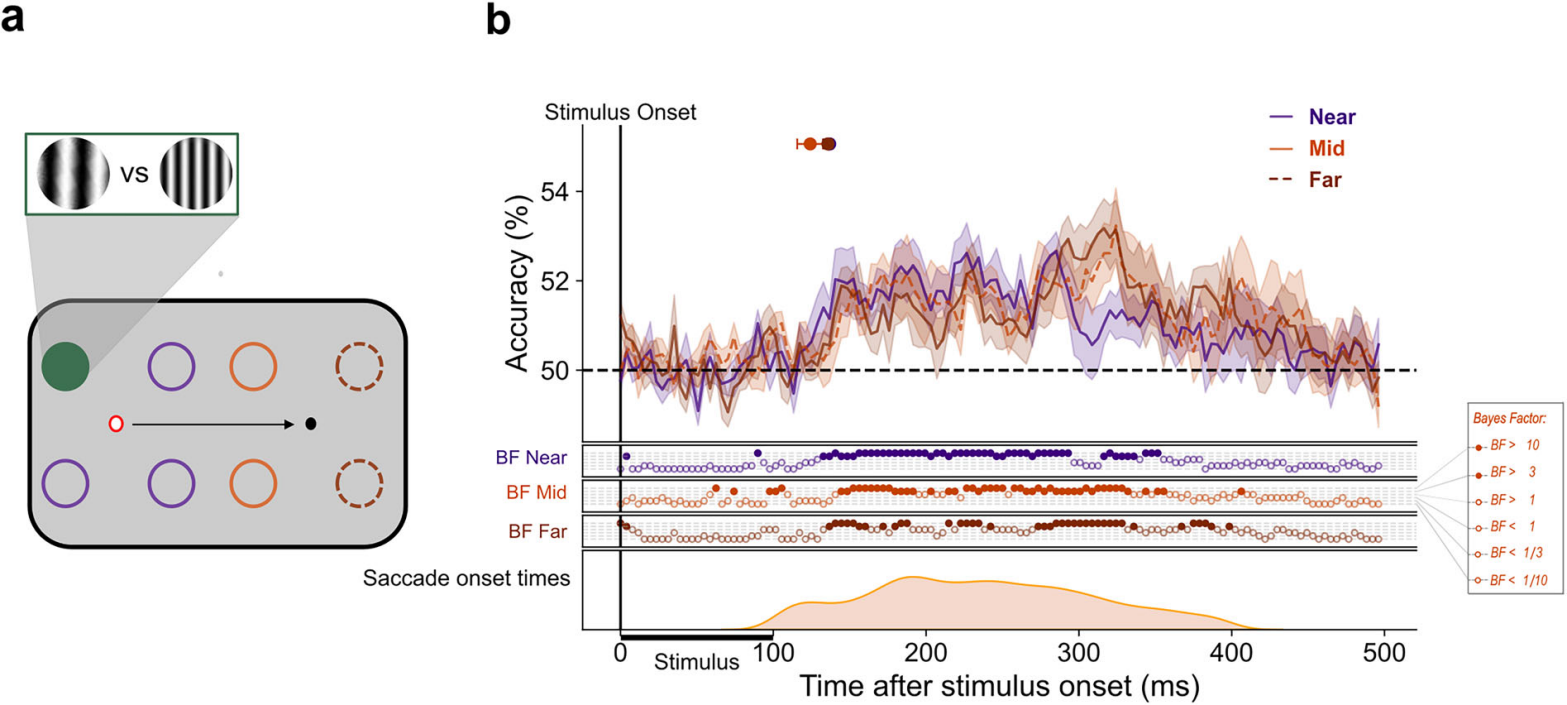
Similar decoding time course across eccentricities in the presence of a saccade. ***a***, Classifiers were trained on high- versus low-frequency stimuli presented at each of the four locations around current fixation (red ring) producing four training sets. Illustrated is an example configuration of train and test locations for classification. The green-filled circle represents the location on which the classifier was trained, and the dark purple (solid line), light orange (solid line), and dark orange (dashed line) indicate the near, mid, and far test locations (during saccade trials), respectively. The red ring signifies current fixation. The gratings illustrate example stimuli at low and high SF. The black arrow represents the saccade. ***b***, The mean classification over time for classifiers trained on fixation trials and tested on near (dark purple), mid (light orange, solid line), and far locations (dark orange, dashed line). The plotted results reflect the average performance at corresponding training and testing timepoints. All decoding results are averaged over eccentricity, across left and right fixation, and subsequently, across subjects. Above-chance decoding onsets are plotted with 95% confidence intervals. The Bayes factors (BF) below the plots indicate the timepoints at which there was substantial evidence in favor of the alternative hypothesis, i.e., decoding above-chance (filled circles). Timepoints at which there was not enough evidence or substantial evidence for the null hypothesis, i.e., chance level, are indicated by open circles. The distribution of saccade onset times is plotted below.

Above-chance decoding of SF for near, mid, and far stimuli first rises above chance at 137 (135–139), 124 (116–133), and 136 (133139) ms, respectively (Fig. 5*b*), with no substantial differences between the decoding time courses. This indicates that during the perisaccadic period after ∼120 ms poststimulus onset, across all stimulus eccentricities, the representation of SF is similar to the parafoveal representation of SF during stable fixation. This is a considerably shorter decoding latency than that found for peripheral stimuli under stable fixation at both mid [207 (190–223) ms] and far [295 (293–297) ms] positions and slightly shorter for near positions [144 (137–151) ms]. Interestingly, the saccade seems to remove latency differences in the spread of SF across eccentricities, giving access to SF information at a similar rate.

## Discussion

In this study, we used MVPA of EEG data to understand if stimulus features are predictively remapped. This method provides a spatiotemporally precise picture of the neural representation of SF, allowing us to characterize the spread of SF information across the visual field during stable fixation. In turn, we determined the extent to which saccade planning provided predictive access to peripheral SF by comparing decoding latencies across fixation and saccade conditions.

We first show an emerging position-invariant representation of SF during stable fixation. We found that, despite training a classifier on SF at parafoveal locations, SF could be decoded from positions up to 20 dva in the periphery. The timing of above-chance decoding was later as eccentricity increased, providing a time course for the spreading of SF information across the visual field. Next, we found that the decoding latency of peripheral SF was accelerated in the perisaccadic period compared with stable fixation, initially suggesting predictive remapping of SF. Interestingly, however, peripheral stimulus SF could be decoded from all parafoveal positions, not just the remapped location, at a similar latency. This indicates that the predictive representation of SF is unbound to a specific retinotopic position. Overall, these results support a predictive representation of peripheral SF due to a saccade which precedes the emergence of a position-invariant representation.

### The spread of spatial frequency across the visual field

We found that the representation of SF spreads gradually over the visual field. This is consistent with stimulus features that are initially processed only by neurons with receptive fields responsive to the stimulus location, but later in a more global fashion across the visual cortex. The early above-chance peak (78 ms), unique to the same location decoding likely signifies the initial retinotopic coding of the stimulus in which SF is bound to its retinotopic position. Once visual input has moved through low-level and mid-level extrastriate areas (V2–V5), stimulus activity begins to spread beyond strictly retinotopic areas to higher-level areas with larger receptive fields (Wurtz and Kandel, 2000). The ability to decode SF at spatial locations adjacent to the trained location (near eccentricity), starting at ∼150 ms after stimulus onset, is consistent with the visual information reaching higher visual areas with larger receptive fields, such as the inferior temporal cortex (Richmond and Wurtz, 1983; Keysers et al., 2001; Hung et al., 2005) that are invariant to changes in object position (Logothetis et al., 1995). The timing of this spatially invariant response also aligns well with human EEG (Liu et al., 2009) and MEG (Fabius et al., 2020) recordings.

Above-chance decoding of SF at peripheral eccentricities emerged at 184 ms (mid) and 297 ms (far) poststimulus onset providing further evidence for the spatially global spread of SF. These results demonstrate that despite large retinotopic eccentricity differences, after ∼290 ms, the stimulus-driven EEG activity is similar across all eccentricities. This could be explained by feature-based attention (FBA), which modulates the firing rate of a neuron tuned to a particular stimulus feature despite it not being directly driven by a stimulus in its RF (Serences and Boynton, 2007). Previous time-resolved research examining the spread of FBA has only used a single stimulus eccentricity, usually positioned within ∼7 dva of fixation with FBA effects emerging at ∼200–280 ms poststimulus onset (Anllo-Vento and Hillyard, 1996; Stoppel et al., 2012; Bartsch et al., 2015, 2017, 2018). We extend this by combining EEG, which provides high temporal resolution, with MVPA, allowing us to pinpoint when neural representations overlap in time, ultimately providing a temporally resolved delineation of the spread of FBA.

### Saccades allow predictive access to peripheral spatial frequency

In line with previous reports of feature remapping, we found that SF could be decoded at the remapped position during the perisaccadic period. Importantly, SF information at that location was available earlier on saccade trials (∼160 ms) compared with the corresponding location on control trials (∼285 ms). This can be considered a predictive response as it occurs at a shorter latency than the typical visual response (Merriam et al., 2007; i.e., cross-decoding the same location in the absence of a saccade). If the availability of SF at the remapped position was the manifestation of saccade-independent feedback processes, such as FBA, we would expect above-chance decoding to emerge at a similar time to control trial decoding given that stimuli overlapped in their retinotopic position. This was not the case. Critically, however, this predictive response was not specific to the remapped location. Instead, the upcoming saccade seems to facilitate predictive access to SF information at all stimulus positions. Unlike under stable fixation, where decoding latency parametrically increased with eccentricity, in the presence of a saccade stimulus, SF could be decoded across the visual field at the same time (∼140 ms) and importantly, before the earliest cross-decoding during fixation (152 ms).

The spatially coarse predictive representation of SF is difficult to reconcile with the precise nature of forward saccadic remapping (Collins et al., 2009); however, it may be explained by convergent remapping, where receptive fields converge toward the saccade target (Tolias et al., 2001; Zirnsak and Moore, 2014). Zirnsak and Moore (2014) reported that when decoding from FEF neurons around the time of a saccade, stimuli were miscategorized with an average error of 7 dva. This aligns with our finding that in saccade trials, SF at both mid and far positions followed a similar decoding time course, suggesting overlapping neural representations. Both mid and far stimuli were positioned at the same distance from the saccade target (5.05 dva). The close proximity of our stimuli to the saccade target and the predictive shift of population RFs (pRF) toward the saccade endpoint, as stated in convergent remapping theories, may mean that mid and far stimuli were similarly encoded. Stimuli at both mid and far positions may have fallen within the shifted pRF, evoking a similar neural response despite different retinotopic coordinates. How exactly convergent remapping contributes to stable vision or the continuity of features across saccades is not known (Marino and Mazer, 2016).

### Could foveal feedback explain the predictive representation of spatial frequency?

Classifiers were trained on SF at four possible retinotopic positions (parafoveal locations); however, all training stimuli extended into the foveal region (Fig. 1*a*), likely eliciting somewhat overlapping representations. Recent work investigating foveal feedback in the presaccadic period found that during saccade preparation, a transient connection is opened between the current and future foveal positions. Using a behavioral detection task, Kroell and Rolfs (2022) found that participants had a higher hit rate for foveal probes when they matched the orientation of the saccade target. Importantly, this effect extended to stimuli up to 6.4 dva around fixation. Interestingly, participants had more congruent false alarms meaning that the saccade target feature was boosted at foveal locations causing participants to “see” it in the noise even when no probe was presented. This suggests an enhancement of the relevant features at foveal locations.

Similarly, we found that peripheral SF could be decoded from the four locations around fixation in anticipation of a saccade. While in our study the stimuli were not the target of the saccade, stimulus SF information may still have been fed back to foveal retinotopic cortex. Previous research, outside the saccade literature, demonstrates that relevant peripheral information is represented at foveal retinotopic cortex (Williams et al., 2008; Fan et al., 2016). The earlier decoding at locations extending into the fovea when a saccade was imminent, may support the idea that feedback connections to foveal retinotopic cortex function to predict stimulus features during saccade preparation (Kroell and Rolfs, 2022).

To better discern the mechanism underlying the predictive neural response reported here, future studies should ensure that training locations are well separated such that stimuli fall either within the parafoveal or foveal region. The parafoveal positions should correspond to the remapped positions of the peripheral stimuli. By more precisely separating training locations, one could check whether peripheral stimulus features are predictively represented only at the exact remapped position, only at the fovea, or more generally across all trained locations.

### Saccade onset time does not modulate the latency of decoding

**Figure 6.**
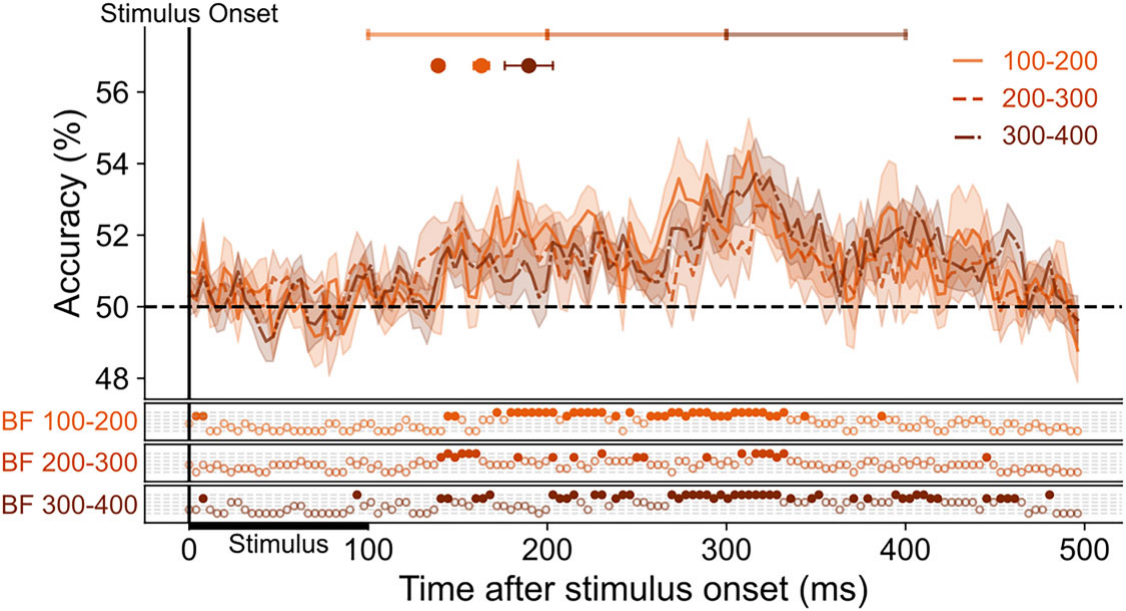
No substantial difference in decoding time course across saccade latencies. The mean classification over time for classifiers trained on fixation trials and tested on saccade trials split into different bins depending on the latency between stimulus onset and saccade onset. The plotted results reflect the average performance at corresponding training and testing timepoints. All decoding results are averaged over locations, across left and right fixation, and subsequently, across subjects. Above-chance decoding onsets are plotted with 95% confidence intervals. The Bayes factors (BF) below the plots indicate the timepoints at which there was substantial evidence in favor of the alternative hypothesis, i.e., decoding above-chance (filled circles). Timepoints at which there was not enough evidence or substantial evidence for the null hypothesis, i.e., chance level, are indicated by open circles.

Despite predictive decoding of SF due to the presence of a saccade, the latency at which SF information spread to parafoveal positions was not modulated by the timing of saccade onset. We split saccade trials into different bins depending on when the saccade occurred after stimulus onset (100–400 ms; Fig. 6) and found that above-chance decoding of peripheral SF at parafoveal positions emerges ∼150 ms, similar to the latency found when combining saccade trials across all bins (main results). This is strange as, if saccade preparation is driving predictive responding, we would expect saccade timing to dictate when this information becomes available. The fact that we see similar results across all bins even those outside the normal saccade preparation range may be explained by the sudden onset of the target stimulus. Increased saccade latencies have been reported when a peripheral stimulus needs to be ignored in order to accurately perform the saccade. While our data cannot reveal if stimulus onset affected saccade latencies as we lack an informative control, previous research has shown that even if the saccade has been prepared, the onset of a peripheral stimulus can prolong the latency of its onset (Dalmaso et al., 2020). This suggests that in the current setup, saccade onset time is not a reliable marker of when saccade preparation begins. Saccade preparation and its associated predictive processes may have begun well before saccade onset, but actual initiation of the saccade is delayed. This may explain the predictive decoding of SF across all bins.

The seeming independence of SF decoding from saccade timing is in stark contrast to spatial information around the time of saccades. Under the same experimental conditions, we found that the remapped location could only be decoded when the stimulus appeared immediately before saccade onset (Moran et al., 2024). Spatial remapping has been shown to be important for the integration of features across a saccade as demonstrated by TMS studies (Prime et al., 2008, 2010). Further research is needed to understand how spatial remapping (Moran et al., 2024) and the predictive anticipation of stimulus features reported here operate in tandem to produce visual stability. One possibility is that spatial remapping and feature-based attention interact to produce the observed results. The preparation of a saccade induces the preactivation of neurons with receptive fields at the remapped location, acting like a location marker (Cavanagh et al., 2010). In combination with this, the grating stimulus captures feature-based attention such that SF is boosted across the screen. Perhaps the spatial pointer at the remapped location and feature-based attention interact to achieve above-chance decoding at locations outside of the stimulus presentation location (Kroell and Rolfs, 2022). However, if this was the case, one might expect higher decoding at the remapped location compared with other irrelevant locations which was not the case.

### Feature-based attention on an unattended feature

The pervasive representation of SF across the visual field in the current experiment is somewhat surprising given that it was task irrelevant. We chose a behavioral task that was orthogonal to the studied feature, to assess automatic remapping of stimulus features and remove any confound of task relevance. However, there is substantial evidence from both single-cell animal recordings (Katzner et al., 2009) and human neuroimaging studies (O’Craven et al., 1999; Schoenfeld et al., 2003; Ernst et al., 2013) to suggest that attention to a single feature can spread to a secondary feature if the features are part of the same object. For example, BOLD responses related to stimulus motion were enhanced even when participants were cued to attend to the color of moving dots (Ernst et al., 2013). Not only does attention spread to the secondary feature, but the task-irrelevant feature can be enhanced across the entire visual field, similar to results reported for attended features (Scholl, 2001). These findings, as well as our own results, support object-based theories of attention in which all features of an attended object are automatically selected for processing, regardless of their task relevance (Duncan, 1984; Scholl, 2001).

An interesting line of investigation for future research would be to compare remapping of task-relevant versus task-irrelevant stimulus features. Previous remapping work has shown that predictive neuronal firing is enhanced when the stimulus to be remapped is a target rather than a distractor (Mirpour and Bisley, 2012; Yao et al., 2016), highlighting the importance of spatial attention for remapping. To the best of our knowledge, there has been no direct comparison of feature remapping for attended versus unattended stimulus features. Whether making spatial frequency task relevant would impact the remapping dynamics reported here is an open question.

### Consequences of spatially invariant representation of spatial frequency

Our results indicate that a global representation of SF emerges after ∼250 ms, potentially indicating that precise remapping of features to the postsaccadic retinotopic location is less urgent than remapping of spatial location. The pervasive representation of SF across the entire visual field calls into question the validity of previous reports of feature remapping. For example, studies that have examined TAE under saccade conditions (Melcher, 2007; He et al., 2018) found that an adaptor, when presented at the postsaccadic retinotopic location of a test stimulus, induced a TAE effect. If, as demonstrated here, features are processed to some degree in a position-invariant manner, then the results reported previously may not be due to a spatially precise remapping of features. This competing hypothesis is particularly likely in studies in which participants have a long stimulus exposure time, giving the spatially invariant representation time to develop. In such a case, neurons tuned to a specific feature are activated across the visual cortex with the adaptor stimulus and so the TAE should be found regardless of its retinotopic position. This is in line with a study that investigated the spread of a motion aftereffect (MAE) across the visual field. They found that an adaptor presented at one location induced an MAE evenly across all other locations tested (Liu and Mance, 2011). Similar results were found when looking at the TAE (Liu and Hou, 2011). This was in the absence of any saccade and so speaks to the global nature of feature-based attention.

Before concluding, we will present some considerations for future research. In the present study, it is difficult to rule out the remapping of visual aftereffects rather than the stimulus itself. Future studies could present a brief mask to obscure any potential visual aftereffects ensuring above-chance decoding reflects remapped stimulus information. In addition, a relatively low number of subjects were collected in the current experiment (*N* = 10). We prioritized a high number of trials within a subject given that classifiers were trained and tested on single-subject data (Teichmann et al., 2022). That said, for some decoding conditions, there was insufficient evidence for either above- or below-chance decoding (BF hovers ∼1) which may be resolved by using a larger sample size.

### Conclusion

We demonstrate an emerging position-invariant representation of SF. In addition, we show a spatially coarse, predictive representation of SF in the presence of a saccade, whereby peripheral stimulus features are encoded earlier. Importantly, this predictive representation is not isolated to the remapped location. This predictive process that allows the visual system earlier access to peripheral stimuli may allow the visual system to maintain a stable representation of the environment and facilitate smooth perception despite frequent eye movements. This is complemented by a spatially invariant representation of stimulus features on saccade landing, creating a robust and flexible system for visual processing.

## References

[B1] Anllo-Vento L, Hillyard SA (1996) Selective attention to the color and direction of moving stimuli: electrophysiological correlates of hierarchical feature selection. Percept Psychophys 58:191–206. 10.3758/BF03211875 8838164

[B2] Bartsch MV, Boehler CN, Stoppel CM, Merkel C, Heinze HJ, Schoenfeld MA, Hopf JM (2015) Determinants of global color-based selection in human visual cortex. Cereb Cortex 25:2828–2841. 10.1093/cercor/bhu078 24770709

[B3] Bartsch MV, Donohue SE, Strumpf H, Schoenfeld MA, Hopf JM (2018) Enhanced spatial focusing increases feature-based selection in unattended locations. Sci Rep 8:16132. 10.1038/s41598-018-34424-5 30382137 PMC6208401

[B4] Bartsch MV, Loewe K, Merkel C, Heinze HJ, Schoenfeld MA, Tsotsos JK, Hopf JM (2017) Attention to color sharpens neural population tuning via feedback processing in the human visual cortex hierarchy. J Neurosci 37:10346–10357. 10.1523/JNEUROSCI.0666-17.2017 28947573 PMC6596623

[B5] Brainard DH (1997) The psychophysics toolbox. Spat Vis 10:433–436. 10.1163/156856897X00357 9176952

[B6] Bruce CJ, Goldberg ME (1985) Primate frontal eye fields. I. Single neurons discharging before saccades. J Neurophysiol 53:603–635. 10.1152/jn.1985.53.3.603 3981231

[B7] Cavanagh P, Hunt AR, Afraz A, Rolfs M (2010) Visual stability based on remapping of attention pointers. Trends Cogn Sci 14:147–153. 10.1016/j.tics.2010.01.007 20189870 PMC2847621

[B8] Chambers CD, Allen CPG, Maizey L, Williams MA (2013) Is delayed foveal feedback critical for extra-foveal perception? Cortex 49:327–335. 10.1016/j.cortex.2012.03.007 22503283

[B9] Collins T, Rolfs M, Deubel H, Cavanagh P (2009) Post-saccadic location judgments reveal remapping of saccade targets to non-foveal locations. J Vis 9:29. 10.1167/9.5.29 19757907

[B10] Dalmaso M, Castelli L, Galfano G (2020) Early saccade planning cannot override oculomotor interference elicited by gaze and arrow distractors. Psychon Bull Rev 27:990–997. 10.3758/s13423-020-01768-x 32607846

[B11] Delorme A, Makeig S (2004) EEGLAB: an open source toolbox for analysis of single-trial EEG dynamics including independent component analysis. J Neurosci Methods 134:9–21. 10.1016/j.jneumeth.2003.10.009 15102499

[B12] Dienes Z (2011) Bayesian versus orthodox statistics: which side are you on? Perspect Psychol Sci 6:274–290. 10.1177/1745691611406920 26168518

[B13] Dimigen O (2020) Optimizing the ICA-based removal of ocular EEG artifacts from free viewing experiments. Neuroimage 207:116117. 10.1016/J.NEUROIMAGE.2019.116117 31689537

[B14] Dimigen O, Sommer W, Hohlfeld A, Jacobs AM, Kliegl R (2011) Coregistration of eye movements and EEG in natural reading: analyses and review. J Exp Psychol Gen 140:552–572. 10.1037/A0023885 21744985

[B15] Dimigen O, Valsecchi M, Sommer W, Kliegl R (2009) Human microsaccade-related visual brain responses. J Neurosci 29:12321–12331. 10.1523/JNEUROSCI.0911-09.2009 19793991 PMC6666125

[B16] Duhamel J, Colby CL, Goldberg ME (1992) The updating of the representation of visual representation. Science 255:90–92. 10.1126/science.1553535 1553535

[B17] Duncan J (1984) Selective attention and the organization of visual information. J Exp Psychol Gen 113:501. 10.1037/0096-3445.113.4.501 6240521

[B18] Engbert R, Kliegl R (2003) Microsaccades uncover the orientation of covert attention. Vision Res 43:1035–1045. 10.1016/S0042-6989(03)00084-1 12676246

[B19] Ernst ZR, Boynton GM, Jazayeri M, Boynton GM (2013) The spread of attention across features of a surface. J Neurophysiol 110:2426–2439. 10.1152/jn.00828.2012.-Contrast 23883860 PMC3841863

[B20] Fabius JH, Fracasso A, Acunzo DJ, van der Stigchel S, Melcher D (2020) Low-level visual information is maintained across saccades, allowing for a postsaccadic handoff between visual areas. J Neurosci 40:9476–9486. 10.1523/JNEUROSCI.1169-20.2020 33115930 PMC7724139

[B21] Fabius JH, Fracasso A, Nijboer TCW, Van Der Stigchel S (2019) Time course of spatiotopic updating across saccades. Proc Natl Acad Sci U S A 116:2027–2032. 10.1073/pnas.1812210116 30655348 PMC6369820

[B22] Fan X, Wang L, Shao H, Kersten D, He S (2016) Temporally flexible feedback signal to foveal cortex for peripheral object recognition. Proc Natl Acad Sci U S A 113:11627–11632. 10.1073/pnas.1606137113 27671651 PMC5068280

[B23] Gibson JJ (1937) Adaptation with negative after-effect. Psychol Rev 44:222–244. 10.1037/h0061358

[B24] Gibson JJ, Radner M (1937) Adaptation, after-effect and contrast in the perception of tilted lines. I. Quantitative studies. J Exp Psychol 20:453. 10.1037/h0059826.

[B25] Golomb JD (2019) Remapping locations and features across saccades: a dual-spotlight theory of attentional updating. Curr Opin Psychol 29:211–218. 10.1016/j.copsyc.2019.03.018 31075621 PMC6776727

[B26] Gordon RD, Vollmer SD, Frankl ML (2008) Object continuity and the transsaccadic representation of form. Percept Psychophys 70:667–679. 10.3758/PP.70.4.667 18556928

[B27] Gottlieb JP, Kusunoki M, Goldberg ME (1998) The representation of visual salience in monkey parietal cortex. Nature 391:481–484. 10.1038/35135 9461214

[B28] Grootswagers T, Robinson AK, Shatek SM, Carlson TA (2024) Mapping the dynamics of visual feature coding: insights into perception and integration. PLoS Comput Biol 20:1–24. 10.1371/journal.pcbi.1011760 38190390 PMC10798643

[B29] Grootswagers T, Wardle SG, Carlson TA (2017) Decoding dynamic brain patterns from evoked responses: a tutorial on multivariate pattern analysis applied to time series neuroimaging data. J Cogn Neurosci 29:677–697. 10.1162/JOCN_A_01068 27779910

[B30] Harrison WJ, Retell JD, Remington RW, Mattingley JB (2013) Visual crowding at a distance during predictive remapping. Curr Biol 23:793–798. 10.1016/j.cub.2013.03.050 23562269

[B31] He T, Fritsche M, de Lange FP (2018) Predictive remapping of visual features beyond saccadic targets. J Vis 18:20. 10.1167/18.13.20 30593063

[B32] Hendrickson A (2005) Organization of the adult primate fovea. In: Macular degeneration (Penfold PL, Provis JM, eds), pp 1–23. Berlin: Springer.

[B33] Herwig A (2015) Linking perception and action by structure or process? Toward an integrative perspective. Neurosci Biobehav Rev 52:105–116. 10.1016/J.NEUBIOREV.2015.02.013 25732773

[B34] Herwig A, Schneider WX (2014) Predicting object features across saccades: evidence from object recognition and visual search. J Exp Psychol Gen 143:1903–1922. 10.1037/a0036781 24820249

[B35] Hollingworth A, Richard AM, Luck SJ (2008) Understanding the function of visual short-term memory: transsaccadic memory, object correspondence, and gaze correction. J Exp Psychol 137:161–181. 10.1037/0096-3445.137.1.163.Understanding PMC2784885 18248135

[B36] Hung CP, Kreiman G, Poggio T, DiCarlo JJ (2005) Fast readout of object identity from macaque inferior temporal cortex. Science 310:863–865. 10.1126/science.1117541 16272124

[B37] Irwin DE, Yantis S, Jonides J (1983) Evidence against visual integration across saccadic eye movements. Percept Psychophys 34:49–57. 10.3758/BF03205895 6634358

[B38] Irwin DE, Zacks JL, Brown JS (1990) Visual memory and the perception of a stable visual environment. Percept Psychophys 47:35–46. 10.3758/BF03208162 2300422

[B39] Jeffreys H (1939) Theory of probability, Ed 1. Oxford: Clarendon.

[B40] Jeffreys H (1961) Theory of probability, Ed 3. New York, NY: Oxford University Press.

[B41] Jonides J, Irwin DE, Yantis S (1982) Integrating visual information from successive fixations. Science 215:192–194. 10.1126/science.7053571 7053571

[B42] Katzner S, Busse L, Treue S (2009) Attention to the color of a moving stimulus modulates motion-signal processing in macaque area MT: evidence for a unified attentional system. Front Syst Neurosci 3:813. 10.3389/neuro.06.012.2009 19893762 PMC2773174

[B43] Keysers C, Xiao D-K, Foldiák P, Perrett DI (2001) The speed of sight. J Cogn Neurosci 13:90–101. 10.1162/089892901564199 11224911

[B44] Kleiner M, Brainard D, Pelli D (2007) What's new in Psychtoolbox-3. Perception 36:1–16.

[B45] Knapen T, Swisher JD, Tong F, Cavanagh P, Zirnsak M, Sprague TC, Pestilli F (2016) Oculomotor remapping of visual information to foveal retinotopic cortex. Front Syst Neurosci 10:54. 10.3389/fnsys.2016.00054 27445715 PMC4915294

[B46] Kroell LM, Rolfs M (2022) Foveal vision anticipates defining features of eye movement targets. Elife 11:e78106. 10.7554/eLife.78106 36082940 PMC9581528

[B47] Lagroix HEP, Yanko MR, Spalek TM (2012) LCDs are better: psychophysical and photometric estimates of the temporal characteristics of CRT and LCD monitors. Atten Percept Psychophys 74:1033–1041. 10.3758/s13414-012-0281-4 22359147

[B48] Liu H, Agam Y, Madsen JR, Kreiman G (2009) Timing, timing, timing: fast decoding of object information from intracranial field potentials in human visual cortex. Neuron 62:281–290. 10.1016/j.neuron.2009.02.025 19409272 PMC2921507

[B49] Liu T, Hou Y (2011) Global feature-based attention to orientation. J Vis 11:8. 10.1167/11.10.8 21920852

[B50] Liu T, Mance I (2011) Constant spread of feature-based attention across the visual field. Vision Res 51:26–33. 10.1016/j.visres.2010.09.023 20887745

[B51] Logothetis NK, Pauls J, Poggiot T (1995) Shape representation in the inferior temporal cortex of monkeys. Curr Biol 5:552–563. 10.1016/S0960-9822(95)00108-4 7583105

[B52] Makeig S, Jung TP, Ghahremani D, Sejnowski TJ (1996) Independent component analysis of simulated ERP data. Technical report INC-9606. Institute for Neural Computation, University of California.

[B53] Marino AC, Mazer JA (2016) Perisaccadic updating of visual representations and attentional states: linking behavior and neurophysiology. Front Syst Neurosci 10:3. 10.3389/fnsys.2016.00003 26903820 PMC4743436

[B54] Mays LE, Sparks DL (1980) Dissociation of visual and saccade-related responses in superior colliculus neurons. J Neurophysiol 43:207–232. 10.1152/jn.1980.43.1.207 6766178

[B55] Melcher D (2005) Spatiotopic transfer of visual-form adaptation across saccadic eye movements. Curr Biol 15:1745–1748. 10.1016/j.cub.2005.08.044 16213821

[B56] Melcher D (2007) Predictive remapping of visual features precedes saccadic eye movements. Nat Neurosci 10:903–907. 10.1038/nn1917 17589507

[B57] Melcher D (2009) Selective attention and the active remapping of object features in *trans*-saccadic perception. Vision Res 49:1249–1255. 10.1016/j.visres.2008.03.014 18457855

[B58] Melcher D, Colby CL (2008) Trans-saccadic perception. Trends Cogn Sci 12:466–473. 10.1016/j.tics.2008.09.003 18951831

[B59] Merriam EP, Genovese CR, Colby CL (2003) Spatial updating in human parietal cortex. Neuron 39:361–373. 10.1016/S0896-6273(03)00393-3 12873391

[B60] Merriam EP, Genovese CR, Colby CL (2007) Remapping in human visual cortex. J Neurophysiol 97:1738–1755. 10.1152/jn.00189.2006 17093130 PMC2292409

[B61] Mirpour K, Bisley JW (2012) Anticipatory remapping of attentional priority across the entire visual field. J Neurosci 32:16449–16457. 10.1523/JNEUROSCI.2008-12.2012 23152627 PMC3508767

[B62] Mirpour K, Bisley JW (2016) Remapping, spatial stability, and temporal continuity: from the pre-saccadic to postsaccadic representation of visual space in LIP. Cereb Cortex 26:3183–3195. 10.1093/cercor/bhv153 26142462 PMC4898672

[B63] Moran C, Johnson PA, Landau AN, Hogendoorn H (2024) Decoding remapped spatial information in the peri-saccadic period. J Neurosci 44:30. 10.1523/JNEUROSCI.2134-23.2024 38871460 PMC11270511

[B64] Morey RD, Rouder JN (2018) Baysefactor: computation of Bayes factors for common designs. Available at: https://philpapers.org/rec/MORBCO-2

[B65] Nakamura K, Colby CL (2002) Updating of the visual representation in monkey striate and extrastriate cortex during saccades. Proc Natl Acad Sci U S A 99:4026–4031. 10.1073/pnas.052379899 11904446 PMC122642

[B66] O’Craven KM, Downing PE, Kanwisher N (1999) fMRI evidence for objects as the units of attentional selection. Nature 401:584–587. 10.1038/44134 10524624

[B67] Paeye C, Collins T, Cavanagh P (2017) Transsaccadic perceptual fusion. J Vis 17:14. 10.1167/17.1.14 28114484

[B68] Parks NA, Corballis PM (2008) Electrophysiological correlates of presaccadic remapping in humans. Psychophysiology 45:776–783. 10.1111/j.1469-8986.2008.00669.x 18513363

[B95] Pelli DG (1997) The VideoToolbox software for visual psychophysics: transforming numbers into movies. Spat Vis 10:437–442. 9176953

[B69] Plöchl M, Ossandón JP, König P (2012) Combining EEG and eye tracking: identification, characterization, and correction of eye movement artifacts in electroencephalographic data. Front Hum Neurosci 6:278. 10.3389/FNHUM.2012.00278/XML/NLM 23087632 PMC3466435

[B70] Prime SL, Vesia M, Crawford JD (2008) Transcranial magnetic stimulation over posterior parietal cortex disrupts transsaccadic memory of multiple objects. J Neurosci 28:6938–6949. 10.1523/JNEUROSCI.0542-08.2008 18596168 PMC6670980

[B71] Prime SL, Vesia M, Crawford JD (2010) TMS over human frontal eye fields disrupts trans-saccadic memory of multiple objects. Cereb Cortex 20:759–772. 10.1093/cercor/bhp148 19641017

[B72] Richmond BJ, Wurtz RH (1983) Visual responses of inferior temporal neurons in awake rhesus monkey. J Neurophysiol 50:1415–1432. 10.1152/jn.1983.50.6.1415 6663335

[B73] Scheel H, Xu R, Jiang N, Mrachacz-Kersting N, Dremstrup K, Farina D (2015) Influence of external cues on synchronized brain-computer interface based on movement related cortical potentials. In: 2015 7th International IEEE/EMBS Conference on Neural Engineering (NER), pp 45–48. IEEE. 10.3389/fnins.2015.00527 PMC4720791 26834551

[B74] Schoenfeld MA, Tempelmann C, Martinez A, Hopf JM, Sattler C, Heinze HJ, Hillyard SA (2003) Dynamics of feature binding during object-selective attention. Proc Natl Acad Sci U S A 100:11806–11811. 10.1073/pnas.1932820100 12960369 PMC208844

[B75] Scholl BJ (2001) Objects and attention: the state of the art. Cognition 80:1–46. 10.1016/S0010-0277(00)00152-9 11245838

[B76] Serences JT, Boynton GM (2007) Feature-based attentional modulations in the absence of direct visual stimulation. Neuron 55:301–312. 10.1016/j.neuron.2007.06.015 17640530

[B77] Sommer MA, Wurtz RH (2002) A pathway in primate brain for internal monitoring of movements. Science 296:1480–1482. 10.1126/science.1069590 12029137

[B78] Stoppel CM, Boehler CN, Strumpf H, Krebs RM, Heinze HJ, Hopf JM, Schoenfeld MA (2012) Spatiotemporal dynamics of feature-based attention spread: evidence from combined electroencephalographic and magnetoencephalographic recordings. J Neurosci 32:9671–9676. 10.1523/JNEUROSCI.0439-12.2012 22787052 PMC6622270

[B79] Subramanian J, Colby CL (2014) Shape selectivity and remapping in dorsal stream visual area LIP. J Neurophysiol 111:613–627. 10.1152/jn.00841.2011 24225538 PMC3921402

[B80] Teichmann L, Moerel D, Baker C, Grootswagers T (2022) An empirically-driven guide on using Bayes factors for M/EEG decoding. Apert Neuro 1:1–10. 10.52294/ApertureNeuro.2022.2.MAOC6465

[B81] Thompson KG, Hanes DP, Bichot NP, Schall JD (1996) Perceptual and motor processing stages identified in the activity of macaque frontal eye field neurons during visual search. J Neurophysiol 76:4040–4055. 10.1152/jn.1996.76.6.4040 8985899

[B82] Tolias AS, Moore T, Smirnakis SM, Tehovnik EJ, Siapas AG, Schiller PH (2001) Eye movements modulate visual receptive fields of V4 neurons. Neuron 29:757–767. 10.1016/S0896-6273(01)00250-1 11301034

[B83] Umeno MM, Goldberg ME (1997) Spatial processing in the monkey frontal eye field. I. Predictive visual responses. J Neurophysiol 78:1373–1383. 10.1152/jn.1997.78.3.1373 9310428

[B84] Walker MF, Fitzgibbon EJ, Goldberg ME (1995) Neurons in the monkey superior colliculus predict the visual result of impending saccadic eye movements. J Neurophysiol 73:1988–2003. 10.1152/jn.1995.73.5.1988 7623096

[B85] Wang X, Zhang C, Yang L, Jin M, Goldberg ME (2023) Perisaccadic and attentional remapping of receptive fields in lateral intraparietal area and frontal eye fields. 1–33.

[B86] Weldon KB, Woolgar A, Rich AN, Williams MA (2020) Late disruption of central visual field disrupts peripheral perception of form and color. PLoS One 15:e0219725. 10.1371/journal.pone.0219725 31999697 PMC6991998

[B87] Wetzels R, Matzke D, Lee MD, Rouder JN, Iverson GJ, Wagenmakers EJ (2011) Statistical evidence in experimental psychology: an empirical comparison using 855 *t* tests. Perspect Psychol Sci 6:291–298. 10.1177/1745691611406923 26168519

[B88] Williams MA, Baker CI, Op De Beeck HP, Shim WM, Dang S, Triantafyllou C, Kanwisher N (2008) Feedback of visual object information to foveal retinotopic cortex. Nat Neurosci 11:1439–1445. 10.1038/nn.2218 18978780 PMC2789292

[B89] Wolf W, Hauske G, Lupp U (1980) Interaction of pre- and postsaccadic patterns having the same coordinates in space. Vis Res 20:117–125. 10.1016/0042-6989(80)90153-4 7434572

[B90] Wurtz RH, Kandel ER (2000) The central visual pathways. In: Principles of neural science (Kandel ER, Schwartz JH, eds), Ed 4, pp 523–547. New York, NY: McGraw-Hill.

[B91] Yao T, Treue S, Krishna BS (2016) An attention-sensitive memory trace in macaque MT following saccadic eye movements. PLoS Biol 14:e1002390. 10.1371/journal.pbio.1002390 26901857 PMC4764326

[B92] Yu Q, Shim WM (2016) Modulating foveal representation can influence visual discrimination in the periphery. J Vis 16:15. 10.1167/16.3.15 26885627

[B93] Zimmermann E, Weidner R, Fink GR (2017) Spatiotopic updating of visual feature information. J Vis 17:6. 10.1167/17.12.6 29049593

[B94] Zirnsak M, Moore T (2014) Saccades and shifting receptive fields: anticipating consequences or selecting targets? Trends Cogn Sci 18:621–628. 10.1016/j.tics.2014.10.002 25455690 PMC4279245

